# Supplementary material for: Improvement of Pseudoalteromonas haloplanktis TAC125 as a Cell Factory: IPTG-Inducible Plasmid Construction and Strain Engineering
Source: Microorganisms. 2020 Sep 24;8(10):1466. doi: 10.3390/microorganisms8101466 (PMC7598627; doi:10.3390/microorganisms8101466)
Supplement: Supplementary file 1 [file microorganisms-08-01466-s001.pdf]

Supplementary material

Improvement of *Pseudoalteromonas haloplanktis* TAC125 as a Cell Factory: IPTG-Inducible Plasmid Construction and Strain Engineering

Andrea Colarusso †, Concetta Lauro †, Marzia Calvanese, Ermenegilda Parrilli and Maria Luisa Tutino *

Dipartimento di Scienze Chimiche, Complesso Universitario Monte Sant'Angleo, Via Cintia 80126 Napoli, Italia; andrea.colarusso@unina.it, concetta.lauro@unina.it, marzia.calvanese@unina.it, erparril@unina.it

* Correspondence: tutino@unina.it; Tel.: +39-081-674317

† These authors equally contributed to this work.

Supplementary Tables

Table S1. Strains used in this study.

Strain	Relevant Traits	Reference or Source
***E. coli* strains**		
DH5α	*supE44, ΔlacU169 (ϕ80 lacZΔM15) hsdR17, recA1, endA1, gyrA96, thi-1, relA1*	Lab stock
S17-1(*λpir*)	*thi, pro, hsd (r− m+) recA::RP4-2-TCr::Mu Kmr::Tn7, Tpr, Smr, λpir*	[14]
***Ph*TAC125 strains**		
*Ph*TAC125 *wt*	Possesses two endogenous plasmids	[9,26]
KrPL	*Ph*TAC125 cured strain without pMtBL plasmid	Lab stock (Unpublished data)
KrPL *lon*	Cured strain without pMtBL plasmid *lon::pVS-lon*	This work
KrPL *lacY+*	Cured strain without pMtBL plasmid *lon::pVS-lacY*	This work

Table S2. Oligonucleotides used in this study.

**Primer name**	**Sequence (5′ - 3′)**	**Application**
p79_fw	GAAATATAGGCATGCACCATGATAACG	Cloning
p79_rv	TGCTCTAGAGTCGACATATGTATTCCTGTTGCATAAACG	Cloning
lonA_SpHI fw	CGGCATGCCCGTAGATGAGCCTGAGC	Cloning
lonA_SacI rv	ACCGAGCTCGTCAATCCCCGAAACC	Cloning
lonB_SacI fw	GCCGAGCTCAGATAGCCGATAGTGCC	Cloning
lonB_EcoRI rv	GTACCGAATTCTGTAACTTGGCCAATACG	Cloning
lonB_SpHI fw	CGGCATGCAGATAGCCGATAGTGCC	Cloning
lonB_HindIII rv	GCGAAGCTTTGTAACTTGGCCAATACGG	Cloning
lonB′_HindIII fw	GCCAAGCTTATAGGTACCAAGGTAAGCTTAGCTAT	Cloning
lonB′_EcoRI rv	GGTACGAATTCCTAACCATCTTTAGGCGTTGCG	Cloning
lon_fw	CCGATCGAGTCGAAATCCCAGT	Screening
lon_rv	GCACTTGGACCATCTTTAGGCG	Screening
lacY_fw	CCACTTAGCCTATTACGCCGTCAGG	Screening
lacY_rv	GTACATATGTATTCCTGTTGCATAATCGAC	Screening
lonY_fw	GCATTACTTGAGGTGTTAGATCCTGAGC	Screening
lonY_rv	CGTTGTCTGGGATCTCTTTTAGGTCACG	Screening

bla_fw	GCAGCAGCCACTGGTAACAGGATTAG	Screening
bla_rv	CGGAGGACCGAAGGAGCTAACCGC	Screening
pheS_fw	ATGTCACATCTCGCAGAACTGG	Screening
pheS_rv	CTGAATTTCATAATCTATTCCTGCC	Screening
R9-gfp_fw	GGAGAGGGTGAAGGTGATGCT	qPCR
R9-gfp_rv	GGTCAGAGTAGTGACAAGTGTTGG	qPCR
lacZ_fw	ATTCGTTGGAGTGATGGCAGTT	qPCR
lacZ_rv	GCGTATTTGGCTTTGCGGTTT	qPCR
PSHA_RS01090_fw	CTAAAGACCAAATCCTTGACGCA	qPCR
PSHA_RS01090_rv	GACCAGCTACCATACCAGCA	qPCR

Table S3. Plasmids used in this study.

**Plasmid**	**Resistance Marker**[1]	**Promoter**	**Purpose**	**Reference**
pMAV-*lacZ*	amp	P*galT*	Expression of *Ph*TAE79 β-galactosidase	[3]
pP79-*lacZ*	amp	P*lacZ*	Expression of *Ph*TAE79 β-galactosidase	This work
p79C-*lacZ*	cam	P*lacZ*	Expression of *Ph*TAE79 β-galactosidase	This work
pMAV-*R9-gfp*	amp	P*lacZ*	Expression of R9-GFP	This work [18]
pP79-*R9-gfp*	amp	P*lacZ*	Expression of R9-GFP	This work [18]
pP79-*pgfp*	amp	P*lacZ*	Expression of a codon optimized gene encoding eGFP	This work
p13C-*lacY*	cam	P13	Expression of *E. coli* lactose permease	This work [1]
pFC-*lacY*	cam	P*aspC*	Expression of *E. coli* lactose permease	This work [21]
pVS-*lon*	amp	none	Mutagenesis of *lon* in KrPL genome	This work [22]
pVS-*lacY*	amp	P13	Mutagenesis of *lon* in KrPL genome	This work [22]

			with the insertion of P13-*lacY* expression cassette	

[1].Amp, ampicillin; cam, chloramphenicol.

Supplementary Figures

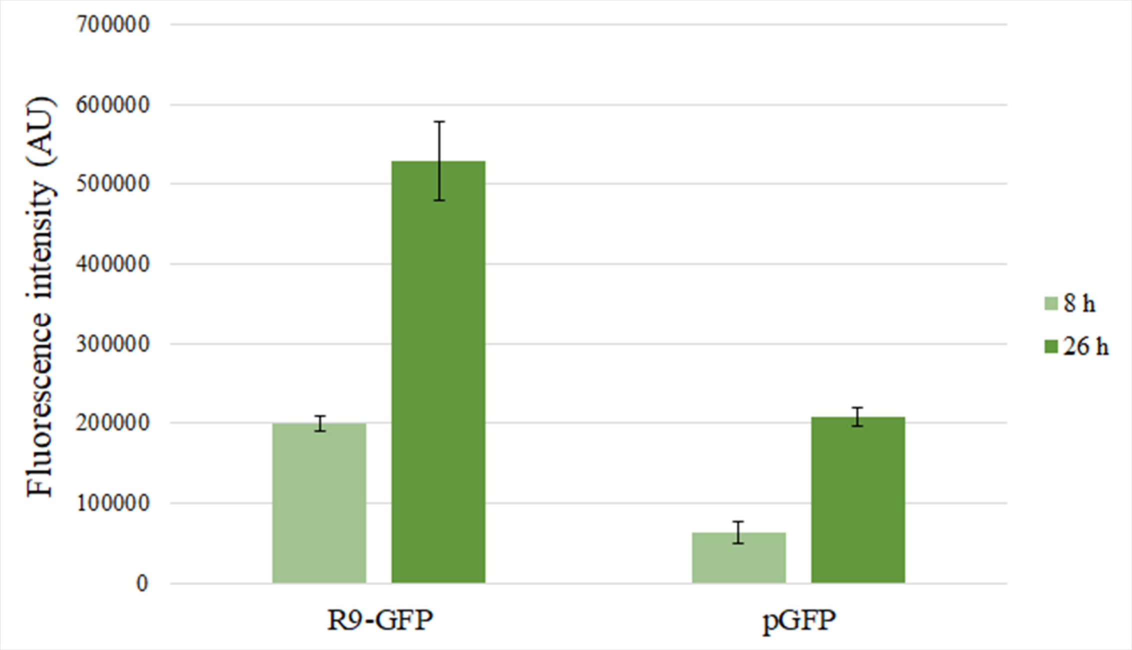

Figure S1. Production levels of R9-GFP and pGFP using pP79 expression plasmid. KrPL recombinant cells were grown in GG at 15 °C and induced with 10 mM IPTG in exponential phase. 8h and 26 h after the induction, the autofluorescence of intact non-recombinant cells was subtracted from the fluorescence of plasmid carrying strains. Fluorescence intensities are reported in arbitrary units (AU) as mean ± SD, $n = 3$.

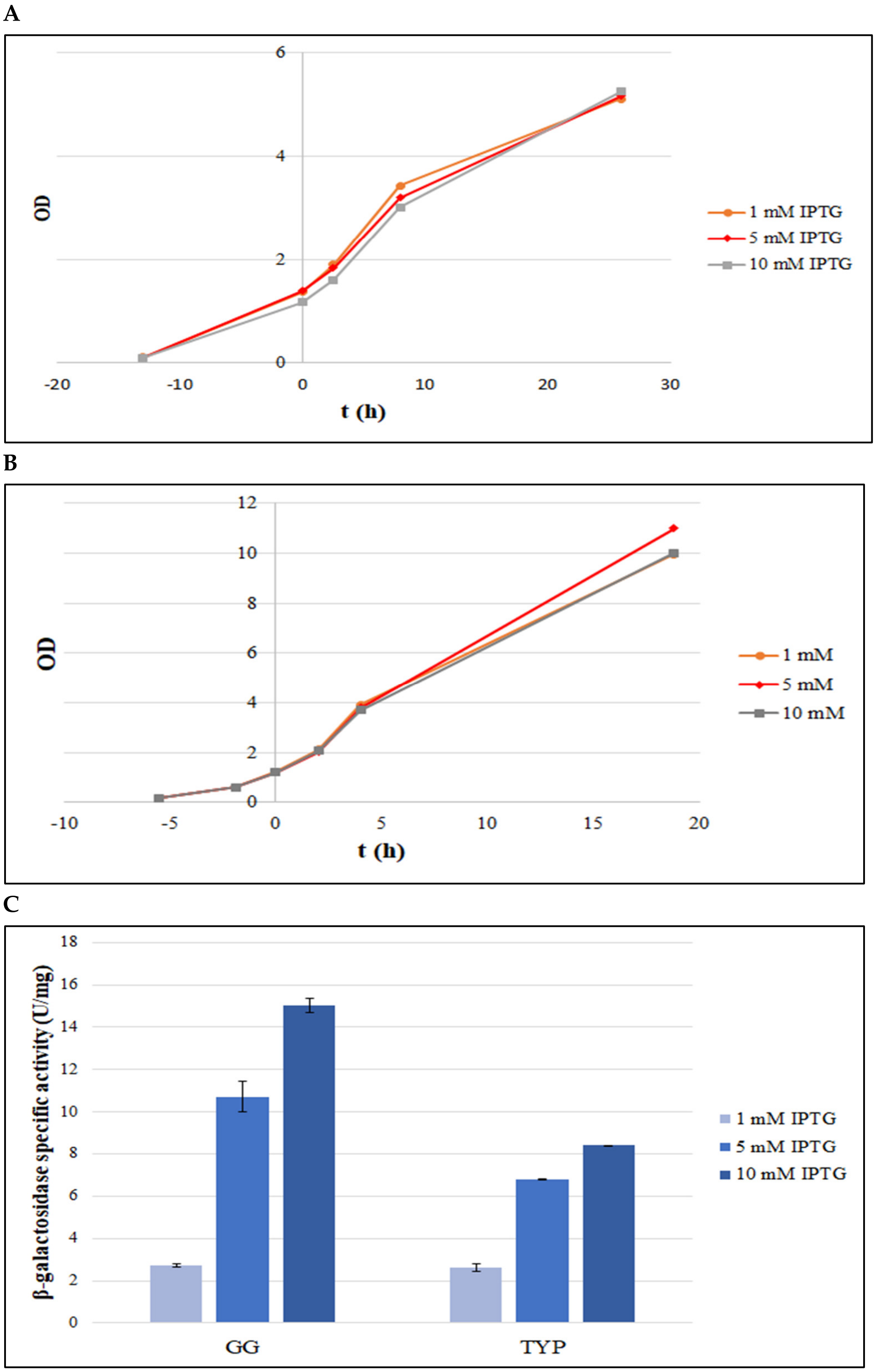

Figure 2. Growths of KrPL pP79-*lacZ* and β-galactosidase production in GG and TYP. The recombinant strains were grown in 250 mL Erlenmeyer flasks filled with either 50 mL GG **(A)** or 50 mL TYP at 15 °C **(B)**. The optical densities were recorded and indicated IPTG quantities were added at time zero (t (h) = 0). **(C)** At the end of the growth experiments, the β-galactosidase specific activities were assayed and are here reported as mean ± SD, n = 3.

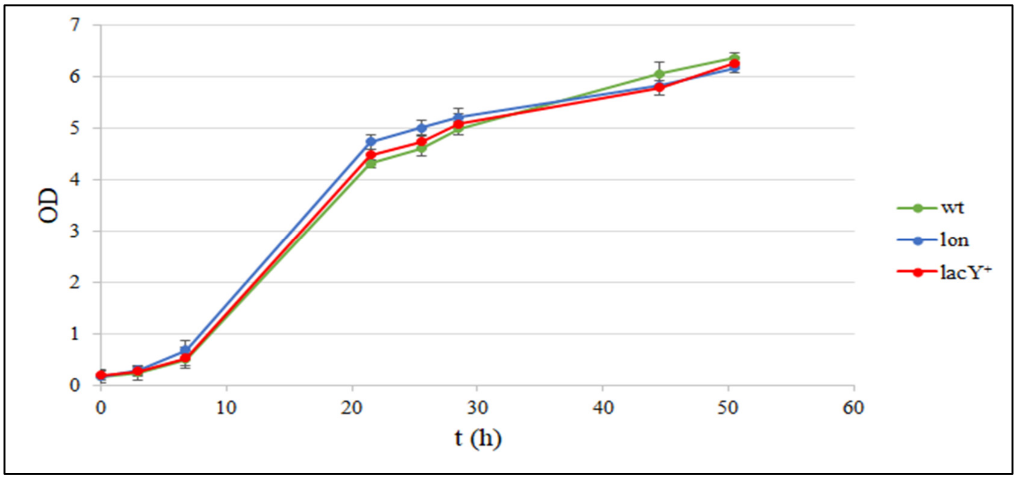

Figure S3. Growths curves of KrPL wt, *lon* and *lacY*⁺ strains. The growths were performed at 15 °C in GG medium. The measures of optical density are expressed as mean ± SD, $n = 2$.
